# Improvement of 1,3-propanediol production from crude glycerol by co-cultivation of anaerobic and facultative microbes under non-strictly anaerobic conditions

**DOI:** 10.1186/s13068-022-02143-9

**Published:** 2022-04-30

**Authors:** Yaqin Sun, Lingyun Liang, Yafeng Zheng, Jindong Han, Zhilong Xiu

**Affiliations:** School of Bioengineering, Dalian University of Technology, No.2 Linggong Road, Ganjingzi District, Dalian, 116024 Liaoning People’s Republic of China

**Keywords:** Synthetic microbial consortium, 1,3-Propandiol, Crude glycerol, *Clostridiumbutyricum*, *Klebsiellapneumoniae*

## Abstract

**Background:**

Natural microbial consortia could efficiently produce 1,3-propanediol (1,3-PDO), a most promising bulk biochemical derived from glycerol that can be used as a monomer in the synthesis of polytrimethylene terephthalate (PTT). While natural microbial communities are made up of a diverse range of microbes with frequently unknown functions, the construction of synthetic microbial consortia allows for the creation of more defined systems with lower complexity.

**Results:**

In this study, the synthetic microbial consortia were constructed by combining facultative microbes of *Klebsiella pneumoniae* DUT2 (KP) and/or *Escherichia coli* DUT3 (EC) cultures with the strictly anaerobic microbe of *Clostridium butyricum* DUT1 (CB) cultures under micro-aerobic conditions. The function of EC and KP during the fermentation process was to deplete oxygen and create an anaerobic environment for CB. Furthermore, KP competes with CB for the consumption of crude glycerol and the production of 1,3-PDO. The interaction of commensalism and competition resulted in the construction of synthetic microbial consortia capable of efficiently converting crude glycerol to 1,3-PDO even under micro-aerobic conditions. In a batch fermentation, the synthetic CB:KP co-culture at an initial abundance ratio of 92.5:7.5, yielded a maximum 1,3-PDO concentration of 52.08 g/L, with a yield of 0.49 g/g and a productivity of 1.80 g/(L.h), which increased by 10%, 9%, and 12%, respectively, when compared to the CB mono-culture under strictly anaerobic conditions. The final 1,3-PDO concentration, yield, and productivity by the synthetic CB:KP consortia increased by 16%, 19%, and 84%, respectively, when compared to the KP mono-culture. At an initial abundance ratio of 85:7.5:7.5, the synthetic CB:KP:EC co-culture achieved the highest 1,3-PDO flux of 49.17%, while 7.43%, 5.77%, 3.15% 4.24%, and 2.13% of flux was distributed to butyric acid, acetic acid, lactic acid, ethanol, and succinic acid pathways. In a fed-batch fermentation, the synthetic CB:KP:EC co-culture demonstrated a maximum 1,3-PDO concentration of 77.68 g/L with a yield of 0.51 g/g which is 30% and 13% higher than the production by the CB mono-culture at 0.02 vvm (nitrogen volume/culture volume/min) N_2_ supply. The initial abundance of CB, which is guaranteed to be at least 85%, enables efficient 1,3-PDO production from crude glycerol via the development of synthetic microbial consortia.

**Conclusion:**

The synthetic microbial consortia demonstrated excellent performance on 1,3-propanediol production under micro-aerobic conditions through the interaction of commensalism and competition. The experimental results demonstrated the potential benefit of using synthetic microbial consortia to produce 1,3-propanediol from crude glycerol.

## Background

Microbial consortia have been widely used in traditional food and beverage fermentation for thousands of years due to their resistance to environmental fluctuations and excellent performance for complex tasks [1, 2]. Natural microbial communities have recently received a lot of attention in the bioconversion of raw materials to biochemicals such as 1,3-propanediol (1,3-PDO), lactic acid, ethanol, and propionate [3–6]. All of these microbial consortia demonstrate excellent utilization of complex carbon sources as well as high efficiency in producing target products.

1,3-Propanediol is a promising bulk biochemical that can be used as a monomer for the synthesis of polytrimethylene terephthalate (PTT) [4, 7–12]. DuPont Tate & Lyle BioProducts and Shenghong Group (China) operate a total 77,000 ton annually capacity in global bio-based 1,3-PDO production [13]. DuPont Tate & Lyle BioProducts and Shenghong Group have developed a 1,3-PDO process with glucose and glycerol as feedstock, respectively. Several studies focusing on natural microbial consortium has been developed to improve raw glycerol metabolism to 1,3-propanediol (1,3-PDO), In fed-batch cultivation, a mixed culture from a municipal wastewater treatment plant produced 70.0 g/L 1,3-PDO with a productivity of 2.6 g/(L.h) [8]. The microbial consortium DL38 having 95.57% abundance of *Klebsiella pneumoniae* achieved a relatively high 1,3-PDO titer of 81.4 g/L with a yield of 0.52 g/g [9]. The effect of initial pH on batch mixed culture fermentation of glycerol was also investigated [10]. The predominant bacteria from *Clostridiaceae*, *Enterococcaceae*, and *Enterobacteriaceae* families produced the highest 1,3-PDO production yield of 0.64 mol/mol at pH 7 and 8. Furthermore, an anaerobic microbial consortium C2-2 M enriched from anaerobic activated sludge with 94.64% abundance of *Clostridium butyricum* produced 60.61 and 82.66 g/L 1,3-PDO in batch and fed-batch fermentation, with productivity of 3.79 and 3.06 g/(L.h), respectively [7]. Performances of continuous fermentations were also investigated by consortium C2-2 M and the highest 1,3-PDO production of 57.86 g/L was achieved with a productivity of 5.55 g/(L.h) at a dilution rate of 0.096 h^−1^ and an initial glycerol concentration of 130 g/L [11]. Furthermore, under micro-aerobic conditions, a novel consortium DUT08 composed primarily of a strictly anaerobic microbe of *C. butyricum*, facultative microbes of *K. pneumoniae* and *E. coli* was able to efficiently convert crude glycerol to 1,3-PDO [4]. In a batch fermentation without nitrogen supply, 43.20 g/L 1,3-PDO was obtained with a yield of 0.39 g/g and a productivity of 0.98 g/(L.h).

Despite the fact that these natural microbial consortia could efficiently convert crude glycerol to 1,3-PDO, there are still significant limitations in answering fundamental ecological and evolutionary questions, as well as the interaction mechanism surrounding natural microbial consortia. Even the most basic natural microbial consortia characterized to date contain tens to thousands of species, making it difficult to experimentally verify which species in such characterizations are actively part of the community or perform key functions [14]. The creation of artificial microbial consortia that retain key features of their natural counterparts is a promising approach to overcoming the challenges associated with studying natural communities. The construction of synthetic microbial consortia allows for the generation of defined systems with reduced complexity.

In our previous work, we isolated 1,3-PDO producing microbes from natural microbial consortium DUT08, including anaerobic *C. butyricum* DUT1 and facultative *K. pneumoniae* DUT2 [4]. In this study, the synthetic microbial consortia was successfully constructed and developed for the production of 1,3-PDO from crude glycerol. Co-culture systems containing a strictly anaerobic microbe of *C. butyricum* and facultative microbes of *K. pneumoniae* and/or *E. coli* were investigated to improve the high oxygen tolerance of the strict anaerobes under micro-aerobic conditions. Furthermore, the distribution of metabolic flux distribution and the abundance of synthetic microbial consortia were discussed.

## Results and discussion

### Comparison of 1,3-PDO production between mono- and co-culture of *C. butyricum* or/and *K. pneumoniae*

*Clostridium butyricum* (CB) and *K. pneumoniae* (KP), each with a significant ability of 1,3-PDO production, have attracted much attention. We chose a CB:KP co-culture in this study to take advantage of these two 1,3-PDO producing microorganisms. *Klebsiella pneumoniae* is a facultative strain that can grow on glycerol under aerobic, micro-aerobic, and anaerobic conditions. As a result, we hope that the growth of *K. pneumoniae* will promote the growth of *C. butyricum* by creating an anaerobic environment. The production of 1,3-PDO using crude glycerol under mono-culture and CB:KP co-culture conditions at the anaerobic serum vial level was tested, and the results are presented in Table 1. *Clostridium butyricum* can grow well with 0.2 vvm (nitrogen volume/culture volume/min) nitrogen supply for 3 min, yielding 10.79 g/L 1,3-PDO with a glycerol yield of 0.54 g/g. In the absence of N_2_, the mono-culture of *C. butyricum* produced no 1,3-PDO. The CB:KP co-culture produced nearly identical amounts of 1,3-PDO at different inoculation ratios. 6.84 g/L 1,3-PDO was produced after 24 h cultivation without N_2_ at the optimal ratio of 3:2. The level of 1,3-PDO production in the CB:KP co-culture was comparable to the KP mono-culture but lower than the CB mono-culture. Many byproducts were produced for the KP mono-culture and the CB:KP co-culture, including acetic acid, lactic acid, succinic acid, and ethanol*.* However, butyric acid was not detected in the CB:KP co-culture. This was interpreted to indicate that dissolved oxygen in the medium inhibited the growth of *C. butyricum* in the CB:KP co-culture, even though *K. pneumoniae* was inoculated to create the anaerobic conditions. *Klebsiella pneumoniae* was primarily responsible for the ability of the CB:KP co-culture to produce 1,3-PDO without N_2_ supply. Under micro-aerobic conditions, *K. pneumoniae* performed worse that *C. butyricum* under anaerobic conditions in producing 1,3-PDO. As a result, the synthetic microbial consortium of *C. butyricum* and *K. pneumoniae* under micro-aerobic conditions demonstrated a weak 1,3-PDO production ability that was inferior to the CB mono-culture under anaerobic conditions.

**Table 1:** Comparison of 1,3-PDO production by the mono-culture and the CB:KP co-culture at different inoculation ratios under anaerobic serum vial level (20 g/L initial glycerol)

Microorganisms	RG (g/L)	1,3-PDO (g/L)	Butyrate (g/L)	Acetate (g/L)	Lactate (g/L)	Succinate (g/L)	Ethanol (g/L)
*C. butyricum* DUT1^a^	0.35 ± 0.12	10.79 ± 0.18	2.49 ± 0.21	–	0.23 ± 0.08	–	–
*C. butyricum* DUT1	19.88 ± 0.34	–	–	–	–	–	–
*K. pneumoniae* DUT3	1.25 ± 0.13	6.72 ± 0.34	–	1.03 ± 0.12	2.16 ± 0.34	0.71 ± 0.13	1.09 ± 0.10
Syn-CB:KP (4:1)	0.78 ± 0.19	6.41 ± 0.19	–	0.56 ± 0.07	1.72 ± 0.15	0.32 ± 0.09	0.89 ± 0.21
Syn-CB:KP (3:2)	1.56 ± 0.23	6.84 ± 0.27	–	0.43 ± 0.11	2.31 ± 0.23	0.34 ± 0.12	0.91 ± 0.07
Syn-CB:KP (2:3)	1.13 ± 0.17	6.62 ± 0.08	–	0.48 ± 0.09	2.41 ± 0.09	0.36 ± 0.10	1.00 ± 0.27
Syn-CB:KP (1:4)	1.27 ± 0.15	6.67 ± 0.34	–	0.56 ± 0.19	2.46 ± 0.21	0.35 ± 0.13	0.82 ± 0.31

^a^Anaerobic conditions: 0.2 vvm N_2_ supply for 3 min

RG, residual glycerol; –, not detectable

### Effect of *E. coli* addition on mono-culture of CB and co-culture of CB and KP for 1,3-PDO production under non-N_2_ aeration conditions

**Fig.1:**
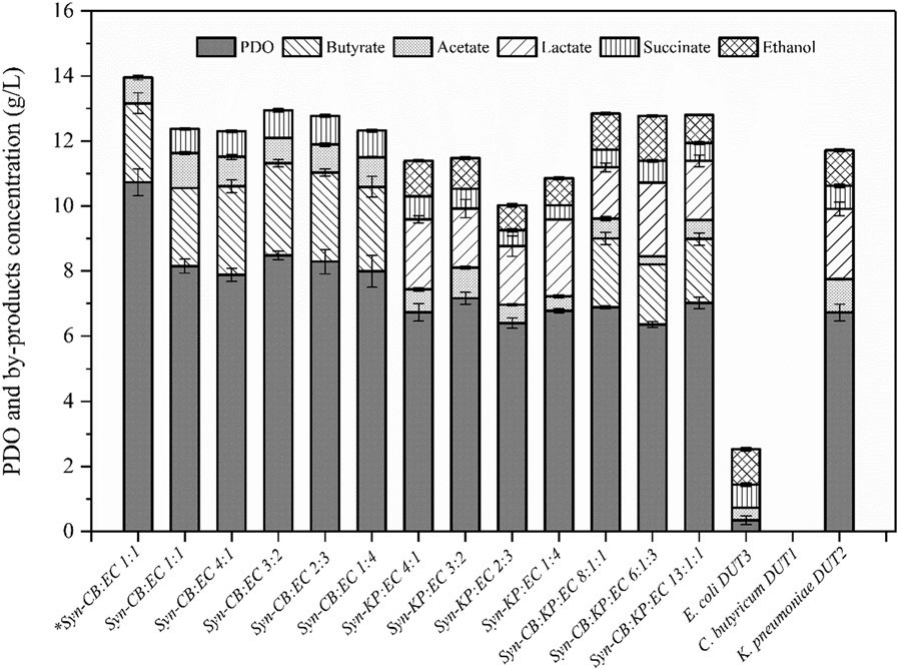
Comparison of 1,3-PDO and byproducts production by the mono-culture and the co-culture at different inoculation ratios without N_2_ supply. The fermentations were carried out at 37 °C and 200 rpm in 250-mL bottle containing 200 mL medium. The fermentations with the KP mono-culture, the CB mono-culture, the synthetic CB:EC co-culture under anaerobic conditions were lasted for 24 h and other groups were 48 h. (*Anaerobic conditions: 0.2 vvm N_2_ supply for 3 min.)

To improve 1,3-PDO production under micro-aerobic conditions, *E. coli* (EC), a microbe that does not produce 1,3-PDO but consumes oxygen, was inoculated to the mono-culture and co-culture cultivation. To that end, the results obtained for 1,3-PDO by different synthetic microbial consortia are represented in Fig. 1.

When compared to the CB mono-culture without N_2_ supply, the results showed that *E. coli* could significantly increase 1,3-PDO production in the CB:EC co-culture at different inoculation ratios. After 24 h of fermentation in the CB mono-culture and the CB:EC co-culture with 0.2 vvm N_2_ supply, glycerol was almost depleted. In the CB:EC co-culture with a 1:1 inoculation ratio and no N_2_ supply, 16.38 g/L glycerol remained in the fermentation media, but only 1.72 g/L 1,3-PDO was produced within 24 h. Glycerol was nearly depleted in the CB:EC co-culture after 48 h of fermentation in the absence of N_2_. In the absence of N_2_, the presence of *E. coli* causes *C. butyricum* to grow and produce 1,3-PDO slowly. When compared to anaerobic conditions (0.2 vvm N_2_ supply), the fermentation time for the CB:EC co-culture was extended under micro-aerobic conditions (no N_2_ supply), and the yield of 1,3-PDO to glycerol decreased by 24% at the inoculation ratio of 1:1. Regardless of the CB:EC inoculation ratios, the CB:EC co-culture could efficiently produce 1,3-PDO once the anaerobic conditions was established by *E. coli.* At a CB:EC inoculation ratio of 3:2, The optimal 1,3-PDO concentration of 8.48 g/L with a yield of 0.42 g/g of glycerol was obtained at a CB:EC inoculation ratio of 3:2. Furthermore, butyric acid was the most abundant byproduct in both the CB mono-culture and the CB:EC co-culture, followed by acetic acid.

The KP:EC co-culture was similar to the metabolic profile of the KP mono-culture. Lactic acid, succinic acid, acetic acid, and ethanol were among the byproducts, with lactic acid having the highest concentration. When EC was inoculated to the CB:KP co-culture, butyric acid and lactic acid were produced as the main byproduct, indicating that anaerobic cultivation conditions were established for good growth of *C. butyricum* in the CB:KP:EC co-culture. The CB:KP:EC co-culture demonstrated a greater ability to remove oxygen due to *E. coli* inoculation than the CB:KP co-culture. As a result, at a CB:KP:EC inoculation ratio of 13:1:1, the highest 1,3-PDO concentration of 7.02 g/L was obtained in the CB:KP:EC co-culture. Because *C. butyricum* and *K. pneumoniae* competed for glycerol to produce 1,3-PDO, the CB:KP:EC co-culture reduced the final 1,3-PDO concentration compared to the CB:EC co-culture. The ability of KP to produce 1,3-PDO is weaker than the ability of CB, and KP is more competitive than CB under micro-aerobic conditions, causing KP to become the dominant in the CB:KP:EC co-culture.

### Metabolic profile and dynamic abundance of synthetic microbial consortia in batch fermentation under non-strictly anaerobic conditions

**Fig. 2:**
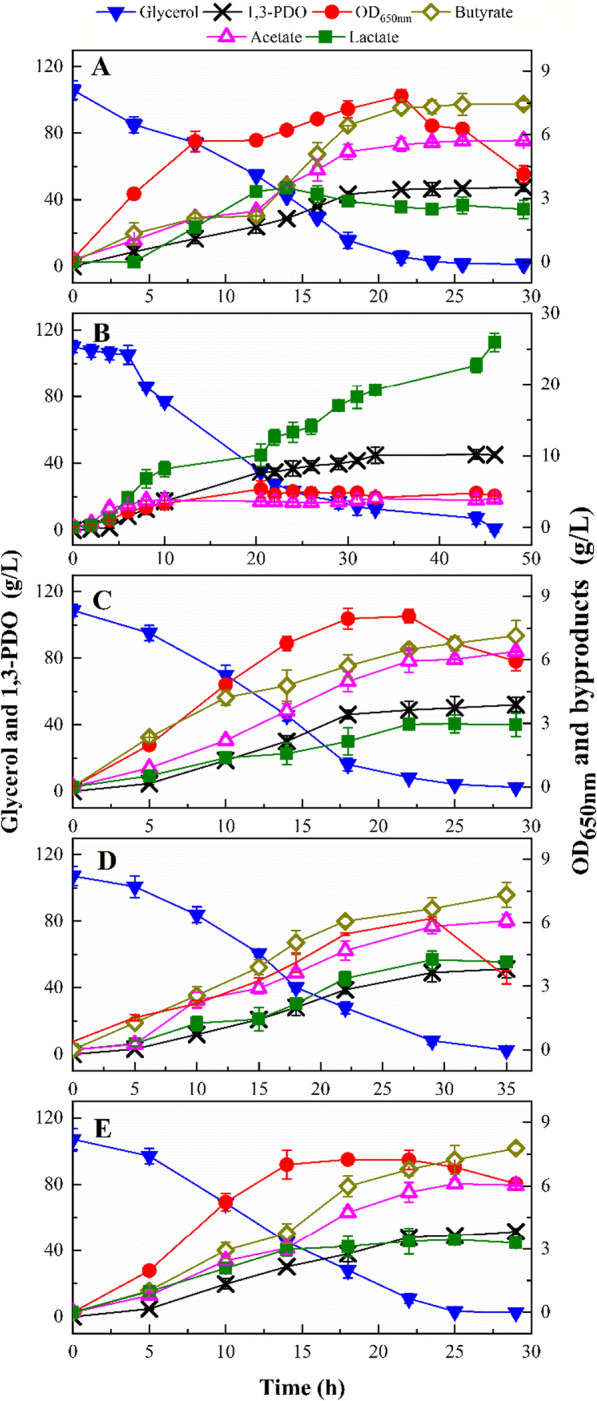
Fermentation performance of mono-culture and synthetic microbial consortia in batch fermentation. **A** The CB mono-culture with N_2_ supply at 0.2 vvm for 3 h; **B** the KP mono-culture without N_2_ supply; **C** the CB:KP co-culture with N_2_ supply at 0.02 vvm for 3 h; **D** the CB:EC co-culture with N_2_ supply at 0.02 vvm for 3 h; **E** the CB:KP:EC co-culture with N_2_ supply at 0.02 vvm for 3 h. The fermentations were carried out at 37 °C, pH 7.0 and 200 rpm in 5-L bioreactor containing 2 L medium

In a previous study, we discovered that the natural microbial consortium DUT08 isolated from wastewater samples could efficiently convert crude glycerol to 1,3-PDO under anaerobic and micro-aerobic conditions. The organism compositions in DUT08 changed during the long-term preservation process due to the coexistence of facultative *K. pneumoniae* and strictly anaerobic *C. butyricum*. The abundance of *C. butyricum* DUT1 decreased from 85.25 (the initial screening) to 60.48% (long-term preservation). Meanwhile, the abundance of *K. pneumonia* DUT2 increased from 0.007 to 38.64%, and the abundance of *E. coli* DUT3 decreased from 12.54 to 0.41%. Furthermore, investigating and determining the function of other unidentified organisms in consortium DUT08, which had a low abundance, was difficult. As a result, the construction and maintenance of a stable, controlled, and robust microbial consortium is critical for efficient 1,3-PDO production. In this case, we constructed and investigated several synthetic microbial consortia composed of strictly anaerobic *C. butyricum*, facultative *K. pneumoniae*, and facultative *E. coli* with or without N_2_ supply to efficiently produce 1,3-PDO. The co-production of 1,3-PDO, butyric acid and lactic acid by the synthetic co-culture in batch fermentation under micro-aerobic and anaerobic conditions was investigated, and the results are shown in Fig. 2.

*Clostridium butyricum* grew well, with no evidence of substrate inhibition. An excellent glycerol consumption rate of 3.55 g/(L.h) was obtained at an initial glycerol concentration of 106.01 g/L and 0.20 vvm N_2_ supply (Fig. 2A). The CB mono-culture produced 47.43 g/L 1,3-PDO with 7.45 g/L butyric acid, 5.73 g/L acetic acid, and 2.48 g/L lactic acid under 0.2 vvm N_2_ supply. The yield and productivity of 1,3-PDO was 0.45 g/g and 1.61 g/(L.h), respectively. Furthermore, *C. butyricum* did not grow under 0.02 vvm N_2_ supply for 3 h at the same initial glycerol concentration, implying that a strictly anaerobic environment did not develop. It has been reported that *C. butyricum* exhibits substrate inhibition at initial glycerol concentration greater than 70 g/L [15]. Dietz et al. observed the same inhibition when mixed cultures were used [8]. In our previous study, obvious substrate inhibition was observed by *C. butyricum* at an initial glycerol concentration of 125.18 g/L [4]. In this study, *C. butyricum* grew well with no substrate inhibition at an initial glycerol concentration of 106.01 g/L and a supply of 0.2 vvm N_2_. However, the cells ceased to grow when supplied with 0.02 vvm N_2_. Once KP and EC were induced into the mono-culture system, the inhibition of dissolved oxygen was alleviated. As a result, it is safe to keep the glycerol concentration below 110 g/L and no dissolved oxygen in medium to achieve a high 1,3-PDO yield and productivity by *C. butyricum*.

The performance of the KP mono-culture was also investigated and the results are presented in Fig. 2B. Although the final 1,3-PDO concentration obtained by the KP mono-culture with an initial glycerol of 110.09 g/L was comparable to that obtained by *C. butyricum*, *K. pneumoniae* demonstrated weaker glycerol consumption ability and lower 1,3-PDO productivity than *C. butyricum*. The fermentation with the KP mono-culture lasted 46 h. The final 1,3-PDO concentration, yield, productivity, and glycerol consumption rate were 45.03 g/L, 0.41 g/g, 0.98 g/(L.h), and 2.37 g/(L.h), respectively. When compared to *C. butyricum*, 1,3-PDO yield, productivity, and glycerol consumption rate by *K. pneumoniae* decreased by about 9%, 39%, and 33%, respectively. Lactic acid was obtained as the main byproduct by *K. pneumoniae* at a concentration of 25.92 g/L. The critical concentration of lactic acid for inhibiting *K. pneumoniae* cell growth was determined to be 19 g/L under anaerobic conditions and 26 g/L under aerobic conditions, respectively [16]. Obvious cell growth inhibition was also observed at 12.59 g/L lactic acid after fermenting for 22 h by *K. pneumoniae* DUT2. Most *K. pneumoniae* produce 2,3-butanediol (2,3-BDO) as a byproduct, and the similarity of 2,3-BDO and 1,3-PDO -causes the separation difficulties [17]. *Klebsiella pneumoniae* DUT2 differs from other *K. pneumoniae* strains in that it does not produce 2,3-BDO during the fermentation process. The dynamic dissolved oxygen and oxidation–reduction potential (ORP) variation of the KP mono-culture during the fermentation process was studied in a 5-L bioreactor. Within 0.5 h, dissolved oxygen was depleted in the KP mono-culture. ORP fell from − 250 to – 580 mv in 8 h, then remained at − 580 mv for 15 h before rising to − 500 mv at the end of fermentation. According to the findings, *K. pneumoniae* scavenged oxygen and could create an anaerobic environment for *C. butyricum* growth in order to produce 1,3-PDO.

**Fig. 3:**
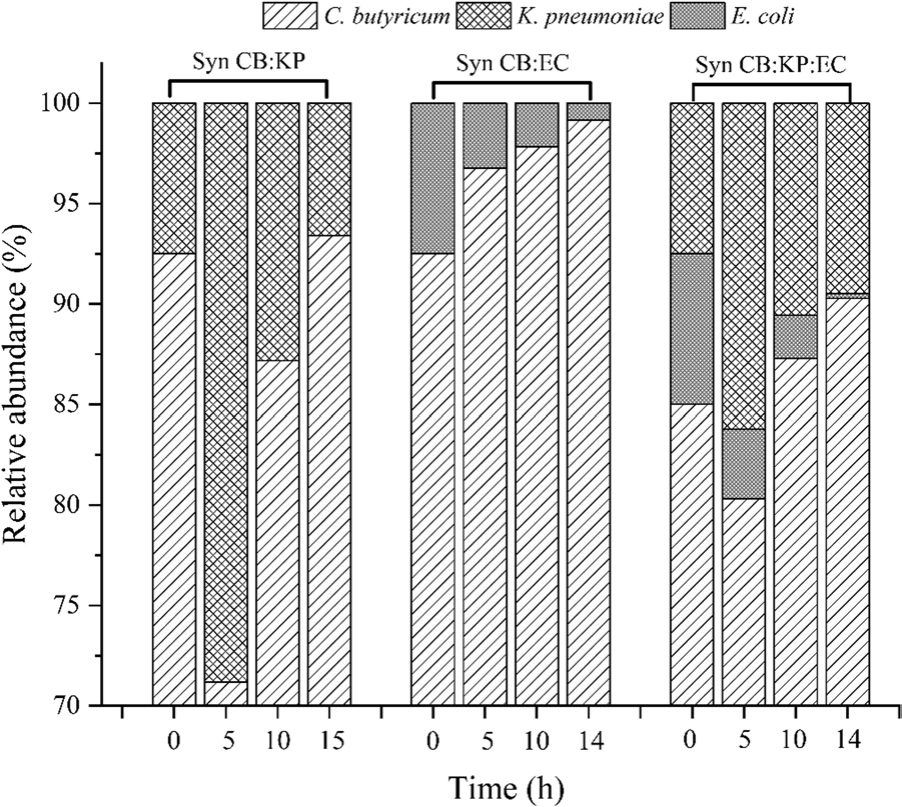
Abundance of synthetic microbial consortia during the batch fermentation at 0.02 vvm N_2_ supply for 3 h. The fermentations were carried out at 37 °C, pH 7.0 and 200 rpm in 5-L bioreactor containing 2 L medium

When KP and CB are co-cultured, they compete for glycerol consumption in order to produce 1,3-PDO, while KP provides an anaerobic environment for CB growth. However, in this study, KP’s ability to produce 1,3-PDO from glycerol was weaker than CB’s. As a result, if the KP abundance in mix culture is too high, the final 1, 3-PDO concentration and glycerol yield will be low. KP abundance should be kept low in order for CB to grow and produce 1,3-PDO efficiently in the CB:KP co-culture. In a previous study, natural microbial consortium DUT08 converted crude glycerol to 1,3-PDO efficiently with an initial abundance of 85.25% *Clostridium*, 12.54% *Escherichia*, and 0.007% *Klebsiella* [4]. As a result, the initial abundance of CB in the synthetic microbial consortia was ensured to be at least 85%. In the CB:KP co-culture with initial inoculation ratio of 92.5:7.5, 52.08 g/L 1,3-PDO with a yield of 0.49 g/g, a productivity of 1.80 g/(L.h) was obtained at an initial glycerol of 108.79 g/L with 0.02 vvm N_2_ supply. Furthermore, byproducts such as 7.14 g/L butyric acid, 2.93 g/L lactic acid, and 6.39 g/L acetic acid were produced (Fig. 2C). Under the same cultivation conditions as the CB mono-culture, no CB growth was observed. CB accounted for 92.50% of the initial abundance, while KP accounted for 7.50% (Fig. 3). Within 5 h, the KP ratio had increased to 28.81%, while the CB ratio had decreased to 71.19%. Following that, CB abundance increased to 87.17% in 10 h and remained at a high level of 93.41% in 14 h.

The dynamic dissolved oxygen and ORP variation of the EC mono-culture during the fermentation process was also investigated. Dissolved oxygen was quickly exhausted in the EC mono-culture under 0.02 vvm N_2_ supply within 10 min. ORP decreased from − 280 to − 590 mv in 4 h, then stayed at – 590 mv for 5 h before increasing to − 400 mv at 12.5 h (20 g/L glucose used). The results showed that *E. coli* scavenged oxygen faster than *K. pneumoniae* and provided an anaerobic environment for *C. butyricum* growth, allowing it to produce 1,3-PDO more quickly. In the CB:EC co-culture with initial inoculation ratio of 92.5:7.5, 51.33 g/L 1,3-PDO with a yield of 0.49 g/g, a productivity of 1.47 g/(L.h) was obtained at an initial glycerol of 107.18 g/L with 0.02 vvm N_2_ supply (Fig. 2D). In comparison to the CB:EC co-culture, KP inoculation in the CB mono-culture can increase glycerol utilization and 1,3-PDO productivity. The abundance of synthetic CB:EC co-culture was also investigated, and the results showed that the abundance of EC decreased from 7.50 to 3.23% in 5 h, and to 0.83% in 15 h. Within 15 h, the abundance of CB increased from 92.5 to 99.17% (Fig. 3).

In a synthetic CB:KP:EC co-culture with initial inoculation ratio of 85:7.5:7.5, 51.43 g/L 1,3-PDO with a yield of 0.49 g/g and a productivity of 1.77 g/(L.h) was obtained at an initial glycerol of 107.18 g/L and a N_2_ supply of 0.02 vvm (Fig. 2E). Furthermore, the main byproducts were 7.77 g/L butyric acid, 6.03 g/L acetic acid, and 3.29 g/L lactic acid, for a total product yield to glycerol of 0.66 g/g. The abundance of synthetic CB:KP:EC, as shown in Fig. 3, indicated that CB was always the dominant bacterium, accounting for more than 80% of the total abundance for the entire fermentation.

**Fig. 4:**
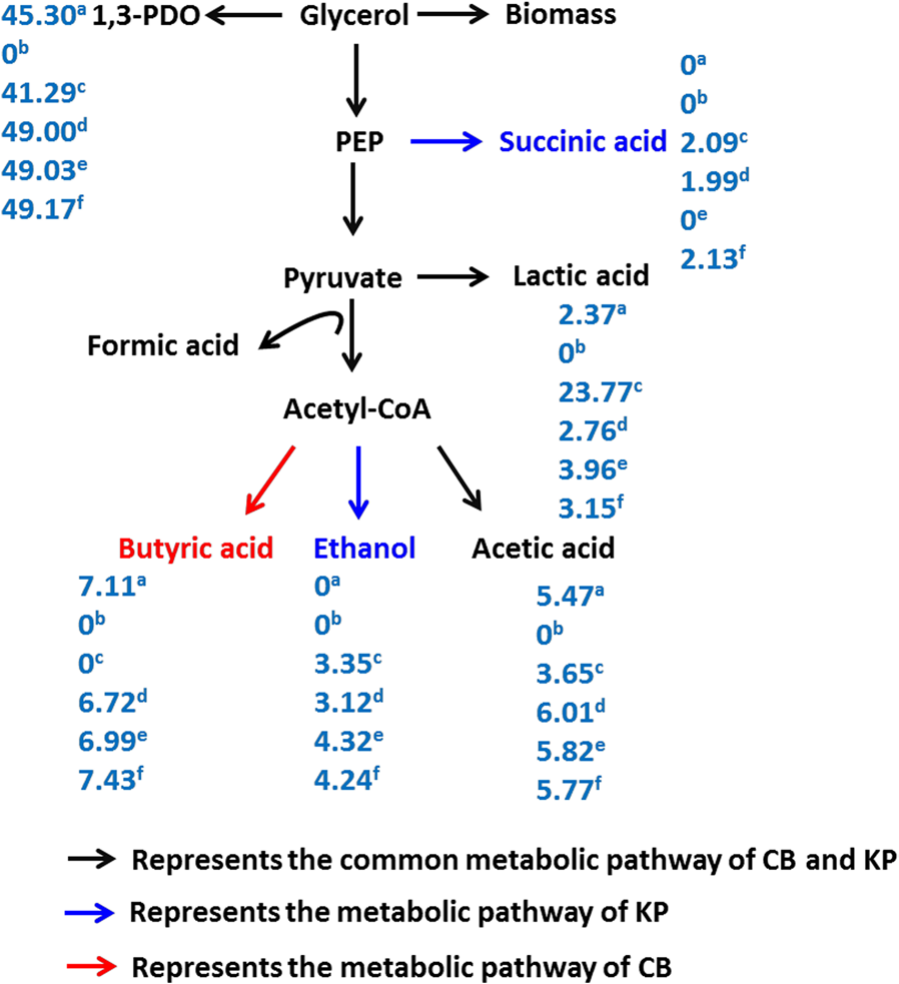
The flux distribution of glycerol metabolism at different mono-culture and synthetic co-culture in batch fermentation. ^a^The CB mono-culture with N_2_ supply at 0.2 vvm for 3 h; ^b^The CB mono-culture with N_2_ supply at 0.02 vvm for 3 h; ^c^The KP mono-culture without N_2_ supply; ^d^The CB:KP co-culture with N_2_ supply at 0.02 vvm for 3 h; ^e^The CB:EC co-culture with N_2_ supply at 0.02 vvm for 3 h; ^f^The CB:KP:EC co-culture with N_2_ supply at 0.02 vvm for 3 h

The metabolic flux distribution of the products was investigated in different cultures, as shown in Fig. 4. For the KP mono-culture, the metabolic flow was mainly distributed to the 1,3-PDO and lactic acid pathways, which accounted for approximately 65.06% of the total glycerol metabolism. Under strictly anaerobic conditions, 52.14% of glycerol metabolic flow was distributed to 1,3-PDO and butyric acid pathways in the CB mono-culture. The results of the synthetic CB:KP, CB:EC, and CB:KP:EC co-cultures demonstrated that under micro-aerobic conditions, strictly anaerobic and facultative bacteria can coexist. The synthetic CB:KP:EC co-culture produced the highest 1,3-PDO flux of 49.17% while 7.43%, 5.77%, 3.15%, 4.24%, and 2.13% of flux was distributed to butyric acid, acetic acid, lactic acid, ethanol, and succinic acid pathways, respectively. All synthetic microbial consortia can efficiently convert crude glycerol to 1,3-PDO and achieve high 1,3-PDO yield. The abundance of 1,3-PDO-producing bacteria with a high 1,3-PDO production capacity, as well as a high glycerol utilization capacity, determines the ability to produce 1,3-PDO. The synthetic CB:KP co-culture achieved a maximum 1,3-PDO concentration of 52.08 g/L in this study at an initial abundance ratio of 92.5:7.5, with a yield of 0.49 g/g and a productivity of 1.80 g/(L.h). The final 1,3-PDO concentration, yield, and productivity of the synthetic CB:KP consortia increased by 10%, 9%, and 12%, respectively, when compared to the CB mono-culture under strictly anaerobic conditions. The final 1,3-PDO concentration, yield, and productivity of the synthetic CB:KP consortia increased by 16%, 19%, and 84%, respectively, when compared to the KP mono-culture. The results showed that the synthetic co-culture established in this study significantly improved 1,3-PDO production from crude glycerol.

### Performance and dynamic abundance of synthetic microbial consortia in fed-batch fermentation under non-strictly anaerobic conditions

**Fig. 5:**
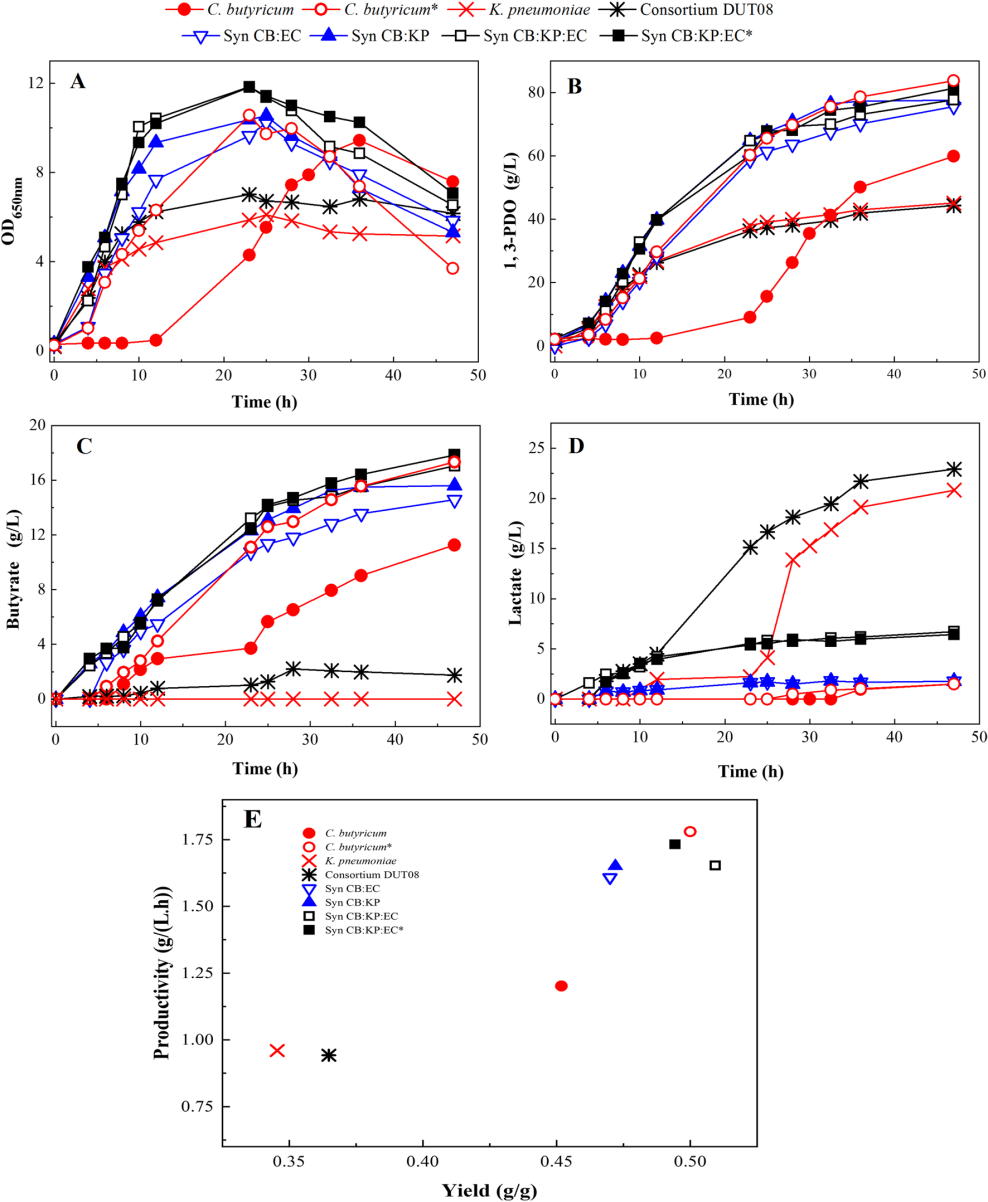
Fermentation performance of the mono-culture and the synthetic microbial consortia at 0.02 vvm N_2_ supply for 3 h under fed-batch fermentation **A** OD_650nm_; **B** 1,3-PDO concentration; **C** butyrate concentration; **D** lactate concentration; **E** yield and productivity. Fermentation was carried out at 37 °C, pH 7.0 and 200 rpm in 5-L bioreactor containing 2 L medium. Residual glycerol controlled around 40 g/L. *Represented 0.2 vvm N_2_ supply for 3 h

A high initial glycerol concentration, as previously stated, inhibits cell growth and reduces glycerol conversion yield. As a result, fed-batch cultivation by various synthetic consortia was developed and compared to evaluate consortia performance. As shown in Fig. 5, obvious cell growth inhibition was observed in the CB mono-culture with 0.02 vvm N_2_ supply, which lasted for at least 12 h. The inoculation of KP and EC resulted in a high cell density in the synthetic CB:KP:EC co-culture. When compared to the synthetic consortia, the natural consortium DUT08 demonstrated similar cell growth to the KP mono-culture and a weak 1,3-PDO production capability. Only 44.29 g/L 1,3-PDO was produced, with a yield of 0.36 g/g and a productivity of 0.94 g/(L.h). The results were very similar to the KP mono-culture, indicating that *K. pneumoniae* has replaced *C. butyricum* as the dominant bacterium in natural consortium DUT08. The final butyric acid concentration of 1.75 g/L and lactic acid concentration of 22.91 g/L obtained by DUT08 also confirmed this deduction. The results presented here are consistent with those presented in the preceding section, indicating that the CB inoculation ratio was an important parameter in the development of synthetic consortia capable of producing 1,3-PDO efficiently from crude glycerol. *Clostridium butyricum* was the dominant bacterium in our previous report, accounting for 85.25% of the proportion in DUT08 [4]. As a result, at an initial glycerol concentration of 117.01 g/L, a higher 1,3-PDO concentration of 58.33 g/L was produced, with a yield of 0.52 g/g and total productivity of 1.94 g/(L.h). Because facultative bacteria and strictly anaerobic bacteria coexist, the preservation conditions for DUT08 are extremely stringent in order to maintain the bacteria ratio. The unstable production of 1,3-PDO is caused by the consortium DUT08’s varied composition. As a result, maintaining a stable and controlled organism composition within a co-culture is critical for efficient 1,3-PDO production.

Synthetic consortia of CB:KP, CB:EC, and CB:KP:EC co-cultures produced the strongest 1,3-PDO formation capability. Among the three constructed synthetic consortia, synthetic CB:KP:EC showed the maximum 1,3-PDO concentration of 77.68 g/L, which is 30% higher than CB mono-culture production at 0.02 vvm N_2_ supply. The synthetic CB:KP:EC consortium obtained a higher 1,3-PDO concentration of 81.39 g/L under 0.2 vvm N_2_ supply. However, when N_2_ supply increased from 0.02 to 0.2 vvm, the 1,3-PDO yield decreased from 0.51 to 0.49 g/g. Butyric acid was the main byproduct when synthetic consortia were used. A synthetic CB:KP:EC co-culture produced more than 17 g/L butyric acid. Furthermore, the synthetic CB:EC co-culture and CB:KP co-culture produced 14.57 g/L and 15.60 g/L butyric acid, respectively. The synthetic consortia are primarily composed of *C. butyricum*, which is producer of butyric acid. The natural consortium DUT08 and the KP mono-culture were used to produce lactic acid as the primary byproduct. Lactic acid concentrations are 22.91 g/L in the natural consortium DUT08 and 20.81 g/L in the KP mono-culture, respectively.

**Fig. 6:**
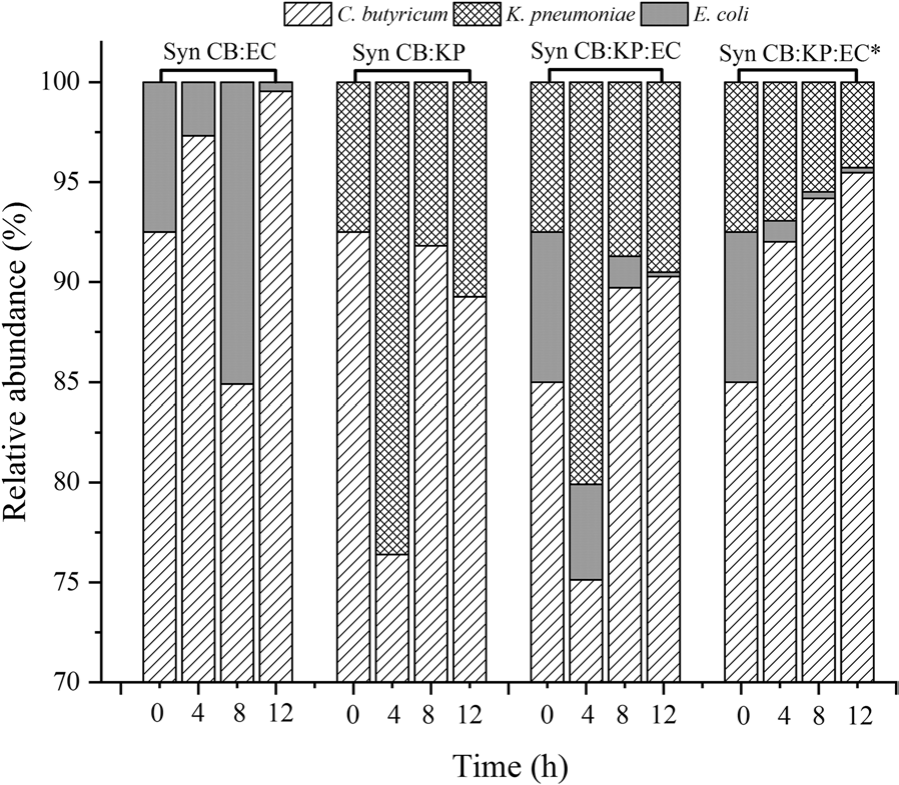
Abundance of synthetic microbial consortia during the fed-batch fermentation process at 0.02 vvm N_2_ supply for 3 h. The fermentations were carried out at 37 °C, pH 7.0 and 200 rpm in 5-L bioreactor containing 2 L medium. Residual glycerol controlled around 40 g/L. *Represented 0.2 vvm N_2_ supply for 3 h

As shown in Fig. 6, the abundance of CB in synthetic CB:EC, CB:KP, and CB:KP:EC co-cultures was 92.5%, 92.5%, and 85.0%, respectively, at the beginning,. Within 4 h, the abundance of KP increased to 23.61% and 20.10%, respectively, in synthetic CB:KP and CB:KP:EC co-culture with 0.02 vvm N_2_ supply. The results showed that CB growth was inhibited for the first 4 h in two synthetic consortia mentioned above. Following that, the abundance of CB increased to 91.82% and 89.71% within 8 h, respectively, and remained high at 89.27% and 90.27% at 12 h. Under 0.02 vvm N_2_ supply, *E. coli* created an anaerobic environment for the CB:EC co-culture in 10 min. As a result, the abundance of CB has always been exceeded than 85%.

Up to date, many attempts have been reported to produce 1,3-PDO from glycerol. Table 2 summarizes 1,3-PDO production by natural 1,3-PDO producers from glycerol in fed-batch fermentations. Among these reported works by pure culture, natural microbial consortia and synthetic consortia, *C. butyricum* DL07 showed the highest 1,3-PDO concentration even with crude glycerol as feedstock. A final yield of 0.52 g/g with a productivity of 2.85 g/(L.h) was obtained. Compared to pure cultures, the natural microbial consortium C2-2 M and synthetic consortium CB:KP:EC in this study also showed excellent performance for converting crude glycerol to 1,3-PDO. As a result, 82.7 g/L and 81.4 g/L 1,3-PDO were obtained by consortium C2-2 M and synthetic consortium CB:KP:EC under anaerobic conditions, respectively. Among these reported studies, natural microbial consortium C2-2 M performed the highest yield and productivity. Synthetic consortium CB:KP:EC retaining key features of their natural counterparts is a promising approach to overcoming the challenges associated with the natural communities. In this study, synthetic consortium CB:KP:EC reserved the characters of high glycerol utilization efficiency by CB, the adaptability of KP and EC to oxygen. As a result, a strictly anaerobic microbe of CB was successfully achieved to cultivate under aerobic conditions and a comparable 1,3-PDO concentration of 77.7 g/L with a yield of 0.51 g/g was obtained.

**Table 2:** The 1,3-PDO production by natural 1,3-PDO producers from glycerol in fed-batch fermentations

Microorganisms	Glycerol source	1,3-Propanediol			Refs.
		Titer (g/L)	Yield (g/g)	Productivity (g/(L.h))	
*C. butyricum* SCUT343-4^a^	Refined	59.2	0.53	2.11	[18]
*C. butyricum* AKR 102^a^	Crude	73.1	0.55	2.48	[19]
*C. butyricum* DL07^a^	Crude	85.5	0.52	2.85	[20]
*C. butyricum* AKR 102^a^	Crude	50.5	0.47	1.80	[21]
*C. butyricum* (Gen 7)^a^	Refined	66.2	0.51	1.38	[22]
*C. butyricum* VPI 1718^a^	Crude	67.9	0.55	0.78	[23]
*C. butyricum* DSM 5431^a^	Crude	70.3	0.68	1.50	[24]
*K. pneumoniae* ATCC 8724	Crude	62.7	0.60	1.74	[25]
*K. pneumoniae* HSL4	Refined	80.1	0.44	2.22	[26]
*K. pneumoniae* LX3^a^	Refined	71.4	0.49	2.24	[27]
*K. pneumoniae* DSM2026^a^	Refined	61.9	0.40	2.00	[28]
*C. freundi*i VK19	Refined	55.6	0.44	0.99	[29]
*C. freundi*i VK19	Crude	47.2	0.38	0.73	[29]
*C. freundi*i FMCC-B294	Crude	68.1	0.40	0.79	[30]
Microbial consortium CJD-S^a^	Crude	41.5	0.34	1.15	[31]
Microbial consortium LS30	Crude	27.8	0.52	0.96	[32]
Microbial consortium C2-2 M^a^	Crude	82.7	0.66	3.06	[7]
Mixed culture	Crude	70.0	0.56	2.60	[8]
Synthetic consortium CB:KP:EC	Crude	77.7	0.51	1.65	This study
Synthetic consortium CB:KP:EC^a^	Crude	81.4	0.49	1.73	This study

^a^Anaerobic conditions

## Conclusions

In this study, the synthetic microbial consortia were successfully constructed by co-culture of three different specified microbes, strictly anaerobic microbe of *C. butyricum* DUT1, and facultative microbes of *K. pneumoniae* DUT2 and *E. coli* DUT3 Different from the strictly anaerobic *C. butyricum*, the synthetic microbial consortia showed low oxygen sensitivity and were able to produce 1,3-PDO under micro-aerobic conditions. In a batch fermentation, the synthetic CB:KP co-culture at an initial abundance ratio of 92.5:7.5, yielded a maximum 1,3-PDO concentration of 52.08 g/L, with a yield of 0.49 g/g and a productivity of 1.80 g/(L.h), which increased by 10%, 9%, and 12%, respectively, when compared to the CB mono-culture under strictly anaerobic conditions. Furthermore, 7.14 g/L butyric acid, 2.93 g/L lactic acid, and 6.39 g/L acetic acid were produced as the main byproducts in the CB:KP co-culture. In a fed-batch fermentation under micro-aerobic conditions, the synthetic CB:KP:EC co-culture showed the maximum 1,3-PDO concentration of 77.68 g/L, which is 30% higher than CB mono-culture. In contrast, a higher 1,3-PDO concentration of 81.39 g/L was obtained by the synthetic CB:KP:EC co-culture under strictly anaerobic conditions. However, the 1,3-PDO yield decreased from 0.51 to 0.49 g/g when cultivation varied from micro-aerobic conditions to strictly anaerobic conditions. In the constructed microbial consortia, the microbes of EC and KP were found to deplete oxygen and provide the anaerobic environment for CB. The investigated results also indicated that CB growth was inhibited for the first 4 h and the abundance of CB exceeded 85% within 12 h. The results demonstrated that the synthetic microbial consortium was an effective and easily controlled cultivation for 1,3-PDO production under micro-aerobic conditions.

## Materials and methods

### Strains and medium

*Clostridium butyricum* DTU1*, K. pneumoniae* DTU2 and *E. coli* DTU3 were isolated from the natural microbial consortium DUT08, which was enriched and isolated from wastewater in the sewage treatment of a petrochemical company [4]. *Clostridium butyricum* DTU1 was anaerobically pre-cultured in a serum bottle containing 100 mL nitrogen-gassed sterilized seed medium at 200 rpm and 37 °C for 12 h. *Klebsiella pneumoniae* DTU2 and *E. coli* DTU3 were aerobically pre-cultured in a serum bottle containing 100 mL sterilized seed medium at 200 rpm and 37 °C for 12 h, respectively. Seed and fermentation media, as well as crude glycerol used were described as our previous report [4]. Crude glycerol used in this study was purchased from Sichuan Tianyu Oleochemical Co. Ltd., China. It contained 78% glycerol, 0.87% ash, 15–17% water, a little salt (the equivalent of electrical conductivity as 0.43% sodium chloride), and pH value was 6.91. The glycerol concentration given in this study indicated the absolute glycerol content of the solution regardless of the impurities.

### Culture conditions

The synthetic microbial consortia were inoculated to the fermentation medium to determine their ability to convert crude glycerol to 1,3-PDO. The co-culture fermentation was performed as follows. The seed culture for CB, KP, and EC was cultivated separately for 12 h. The optical density at 650 nm for CB and KP was about 2.5 and for EC about 1.5. The total inoculation volume of synthetic consortium for co-culture was 10% (v/v) for shake flask and bioreactor. The inoculation ratio of synthetic consortium refers to the inoculation volume ratio for each microorganism. The OD value for the synthetic seed culture was different with different inoculation ratios. To keep a constant initial OD for fermentations, the synthetic seed cultures were diluted to a similar OD value.

Shake-flask culture was carried out in 250-mL serum bottles with medium volume of 200 mL at 37 °C and 200 rpm. The fermentations with the KP mono-culture, the CB mono-culture, the synthetic CB:EC co-culture under anaerobic conditions were lasted for 24 h, and other groups were 48 h. Three replicates were performed for shake-flask cultures.

The batch and fed-batch fermentations were carried out in a 5-L bioreactor with the working volume of 2 L at 37 °C and 200 rpm. The pH was automatically set to 7.0 by adding 5 mol/L NaOH. When the residue glycerol concentration was less than 20 g/L in fed-batch fermentations, continuous feeding was initiated, and the feeding rate was adjusted to keep the glycerol concentration between 20 and 30 g/L. Anaerobic fermentation was performed for 1 h before and 2 h after inoculation with 0.2 vvm N_2_ supply. Micro-aerobic fermentation was achieved with or without 0.02 vvm N_2_ supply for 1 h before and 2 h after inoculation. Samples were taken at regular intervals to analyze the concentrations of biomass, glycerol, and products. Two replicates were performed for the batch and fed-batch fermentations. The metabolic flux distributions of the products were calculated by dividing the product formation rates (qp) by the glycerol uptake rate (qs) and multiplying by 100.

### qPCR analysis

Quantitative real-time PCR was used to examine the bacterial compositions of synthetic microbial consortia. The manufacturer’s instructions were followed when extracting genomic DNATaKaRa MiniBest Bacterial Genome DNA Extraction Kit Ver. 3.0). Samples were taken at regular intervals from batch and fed-batch fermentation (5-L bioreactor containing 2 L medium, 37 °C, pH 7.0 and 200 rpm). The purity of DNA was checked by using a NanoDrop ND-2000 spectrophotometer (NanoDrop technologies, Wilmington, DE) and electrophoresis. Primers were designed based on the differences in 16S rRNA sequences among *C. butyricum* DUT1, *K. pneumoniae* DUT2, and *E. coli* DUT3 listed in Table 3.

**Table 3:** Primers designed for *C. butyricum* DUT1, *K. pneumoniae* DUT2 and *E. coli* DUT3

Primer	Sequences(5′–3′)	Expected size (bp)	Targeted strain
*HgF*	AAGAAGCTTTAGAAGATCCTAA	261	*C. butyricum* DUT1
*HgR*	GGACAACATGAGGTAAACATTG	261	*C. butyricum* DUT1
*phoEF*	TGCCCAGACCGATAACTTTA	210	*K. pneumoniae* DUT2
*phoER*	CTGTTTCTTCGCTTCACGG	210	*K. pneumoniae* DUT2
*ybbWF*	TGATTGGCAAAATCTGGCCG	210	*E. coli* DUT3
*ybbWR*	GAAATCGCCCAAATCGCCAT	210	*E. coli* DUT3

The reaction was performed in a final volume of 20 μL containing 10 μL of SYBR Premix EX Taq (1X) (TaKaRa Biotechnology Co., Dalian, China), 0.8 μL of each primer (0.4 μM), 0.4 μL of ROX Reference Dye II (1X), and 2 μL of genomic DNA (1 ng/μL).

The PCR was performed using the following protocol: 95 °C for 30 s, followed by 40 cycles at 95 °C for 3 s, and 60 °C for 30 s. For each sample, the assay was performed at least three times. Three standard plasmids were used to generate standard curves for quantification. Standard plasmid was constructed by pESI-T vector ligation with PCR product of 16S rRNA, and then transforming it into *E. coli* DH5α. The copy number was calculated as follows:

$$ {\text{Copy}}\,{\text{number}} = \frac{{{\text{DNA}}\,{\text{amount}}}}{{{\text{DNA}}\,{\text{amount}} \times 660 \times 1 \times 10^{9} }} \times 6.022 \times 10^{23} . $$

qPCR reactions were run on serial dilutions of each standard plasmid to correlate the threshold cycle number (Ct value) to copy numbers of the target sequence and to generate standard curves for quantification in unknown samples. Standard curves were linear across five orders of magnitude of 10^2^–10^7^ copies with R^2^ of 0.99.

### Analytical methods

Biomass concentration was determined by the measurement of optical density at 650 nm (OD_650_). Glycerol and main products (1,3-PDO, butyric acid, acetic acid, lactic acid, succinic acid, and ethanol) were analyzed using HPLC (Waters 600E) equipped with an Aminex HPX-87H column with a column temperature of 65 °C [4]. The mobile phase was 5 mM H_2_SO_4_ with a flow rate of 0.6 mL/min. The samples for HPLC were centrifuged at 9720×*g* for 10 min and the supernatant was used for analysis. Before injection, sample solutions were diluted to appropriate concentrations and filtered through a 0.22-μm membrane filter. All data presented are the average of two or three independent experiments performed under the same culture conditions.

## Data Availability

The datasets used and/or analyzed during the current study are available from the corresponding author on reasonable request.

## Abbreviations

1,3-PDO: 1,3-Propanediol
CB: *Clostridium butyricum* DUT1
KP: *Klebsiella* *pneumoniae* DUT2
EC: *Escherichia** coli* DUT3
PTT: Polytrimethylene terephthalate
RG: Residual glycerol
2,3-BDO: 2,3-Butanediol
